# Lossy Image Compression in a Preclinical Multimodal Imaging Study

**DOI:** 10.1007/s10278-023-00800-5

**Published:** 2023-04-10

**Authors:** Francisco F. Cunha, Valentin Blüml, Lydia M. Zopf, Andreas Walter, Michael Wagner, Wolfgang J. Weninger, Lucas A. Thomaz, Luís M. N. Tavora, Luis A. da Silva Cruz, Sergio M. M. Faria

**Affiliations:** 1 Instituto de Telecomunicações, Morro do Lena—Alto do Vieiro, Leiria, Portugal; 2 University of Coimbra, Coimbra, Portugal; 3 Vienna BioCenter Core Facilities GmbH, 1030 Vienna, Austria; 4 Ludwig Boltzmann Institute for Experimental and Clinical Traumatology Vienna, Vienna, Austria; 5 Centre of Optical Technologies, Aalen University, Aalen, Germany; 6 Institute of Applied Research, Aalen University, Aalen, Germany; 7 Division of Anatomy, Center for Anatomy & Cell Biology, Medical University of Vienna, Vienna, Austria; 8 School of Technology and Management, Polytechnic of Leiria, Morro do Lena—Alto do Vieiro, Leiria, Portugal; 9 Department of Electrical and Computer Engineering, University of Coimbra, Coimbra, Portugal; 10 Instituto de, Telecomunicações University of Coimbra, Coimbra, Portugal

**Keywords:** Biomedical imaging, Image coding, Image segmentation, Performance evaluation

## Abstract

The growing use of multimodal high-resolution volumetric data in pre-clinical studies leads to challenges related to the management and handling of the large amount of these datasets. Contrarily to the clinical context, currently there are no standard guidelines to regulate the use of image compression in pre-clinical contexts as a potential alleviation of this problem. In this work, the authors study the application of lossy image coding to compress high-resolution volumetric biomedical data. The impact of compression on the metrics and interpretation of volumetric data was quantified for a correlated multimodal imaging study to characterize murine tumor vasculature, using volumetric high-resolution episcopic microscopy (HREM), micro-computed tomography (µCT), and micro-magnetic resonance imaging (µMRI). The effects of compression were assessed by measuring task-specific performances of several biomedical experts who interpreted and labeled multiple data volumes compressed at different degrees. We defined trade-offs between data volume reduction and preservation of visual information, which ensured the preservation of relevant vasculature morphology at maximum compression efficiency across scales. Using the Jaccard Index (JI) and the average Hausdorff Distance (HD) after vasculature segmentation, we could demonstrate that, in this study, compression that yields to a 256-fold reduction of the data size allowed to keep the error induced by compression below the inter-observer variability, with minimal impact on the assessment of the tumor vasculature across scales.

## Introduction

The field of bioimaging is experiencing several major boosts: for single cell imaging, the resolution revolution in electron cryo-microscopy [1] led to an exponential increase in resolved single particle structures. This is accompanied by a volumetric resolution which allows to image entire cells in 3D at a resolution of a few nanometers using, for example, focused ion beam scanning electron microscopy. The same holds true for advanced light microscopy that routinely resolves subcellular structures, both below the diffraction limit (super-resolution) and in thick tissue with increased penetration depth (light-sheet, multiphoton, or high-resolution episcopic microscopy—HREM) [2]. Additionally, frontiers of bioimaging are currently being pushed towards the integration and correlation of several modalities to tackle biomedical research questions holistically and across scales (correlative multimodal imaging—CMI) [3]*.* CMI is specifically moving towards the integration of in vivo with subsequent ex vivo imaging modalities to bridge the gap between preclinical and biological imaging [4, 5]. Prominent examples of preclinical imaging include micro-computed tomography (µCT) and micro-magnetic resonance imaging (µMRI) since they allow one to acquire 3D high-resolution datasets without violating the integrity of the specimen, making them a non-destructive alternative to serial histology [6]. While, traditionally, µCT was used to assess bone morphology and density, recently, its range of applications — based on radiopaque contrast agents — has expanded towards the characterization of heart, kidney, liver and lung morphometry and function, vascular analysis, quantification of fat tissue, and the assessment of tumor load in organs. This information can be substantially enriched using µMRI which gathers metabolic, physiological, and functional information non-destructively at a resolution of a few micrometers. Due to its non-ionizing interaction with the net nuclear magnetic moment of nucleons, in contrast to µCT, repeated studies are feasible, with no known side effects, to monitor physiological and pathophysiological processes in vivo, including ageing or disease progression.

As a result of these technological evolutions, it is becoming routine to collect raw images of hundreds of gigabytes per imaging session. Recent advances in cryo-EM, including those in detector technology with faster frame rates, have substantially increased data generation, with a typical single particle imaging session acquiring data in the gigabyte range. Light-sheet microscopy allows integrating super-resolution modules with lattice light-sheet microscopy acquiring data in the range of terabytes for a single 4D data set. A single HREM session can produce a dataset of several GB due to its micrometer resolution and capability of visualizing relatively big volumes of several mm^3^, and the volume of data of complex features and broad dimensions acquired in biomedical imaging, including µMRI and µCT, is growing exponentially [7]*.* In combination with dynamic CMI approaches, the sheer size of the bioimaging data truly enters the big data regime. Indeed, the biggest bottleneck of CMI is currently data handling and storage due to the plethora of complex, multimodal, time-varying, and diverse volumetric imaging data. Further increases in throughput and automation will generate even more data and raise the pivotal question of how to handle these huge amounts of data. On top of this, there are currently no data handling guidelines or data retention and management plans. Submission of acquired image data to public archives (EMPIAR or cell-IDR) will only allow to store a fraction of the acquired data [8]. In addition to the growing size of bioimage datasets, the diversity of image modalities included in multimodal workflows make the management and analysis of the data more complex [9]. One approach to alleviate this challenge is data compression. Since many quantitative analyses of image datasets do not exploit the full resolution of the acquisition scheme but are focused on specific macro-structures that are not greatly affected by compression artifacts, even lossy compression schemes might represent a versatile approach in tackling the huge amount of data in bioimaging.

An image compression system encompasses an encoder and a decoder: the encoder block is responsible for reducing image data redundancy and encoding this information into a compressed file (bitstream); a decoder block is responsible for decoding a bitstream and decompressing the image data, so that an image can be reconstructed and used for various purposes. The greatest advantage of lossless compression algorithms is their reversibility, i.e., lossless compressed image data can be fully restored without any mathematical difference. Contrarily, lossy image compression introduces irreversible changes to the image data, which induces mathematical differences between the original and the decompressed data. Another difference between these two classes of image compression systems is their efficiency: while standard lossless image compression typically delivers compression ratios in the order of 2:1 [10], lossy compression can achieve much higher compression rates at the expense of some distortion. By archiving compressed bitstreams instead of the raw image data, more images can be stored, and less bandwidth is required to transmit them. Both are urgently needed in the bioimaging community, including the multitude of bioimaging facilities.

In this paper, based on a published CMI feasibility study to visualize murine tumor vasculature across scales [11], we (i) compare achievable compression rates and data storage reduction for each modality using lossy compression, (ii) assess the minimal rate to preserve relevant quantitative image content (in this case, the tumor vasculature), and (iii) specifically quantify compression schemes for the microscopy technique HREM, and the preclinical imaging modalities µCT and µMRI.

### JPEG2000 and H.265/HEVC — Overview

JPEG2000 is an ISO standardized waveform-based coding system [12] built-upon the discrete wavelet transform (DWT). It provides features such as resolution scalability (i.e., the ability to restore the full-resolution image data passing through multiple down-sampled versions of the signal), partial decoding (i.e., the ability to decode specific portions of the image independently—random access), and region-of-interest coding (i.e., the ability to encode predefined regions with higher quality, which are transmitted first in the bitstream). Furthermore, extensions such as the multi-component transform (MCT) that expands the proposed single-component compression for polychrome content, or the 3D DWT version [13] to exploit the volumetric redundancy that biomedical sequences usually present, make this coding system versatile enough to compress N-dimensional (*N* ≥ 3) image sequences.

The JPEG2000 coding pipeline includes multi-component transformation, tiling, wavelet-transform, quantization, and entropy-coding. The multi-component transformation module makes use of either fixed non-integer (lossy) or integer (lossless) transforms to decorrelate the spectral components of the input image. The tiling module aims to divide each component into tiles (i.e., sub-units of data) which undergo independent compression; therefore, the tile dimension is related with the granularity of the random access achieved. An L-Level wavelet transform [12] is then separately applied to each component of a tile, generating a set of sub-bands of wavelet coefficients associated with varying degrees of texture details. The lossless mode uses a [3, 5] biorthogonal filter and the lossy mode employs a [7, 9] biorthogonal filter, as their wavelet base filters. In the case of lossy compression, the wavelet coefficients can be quantized differently in each sub-band. Then, the resulting DWT coefficients are grouped into non-overlapping rectangular structures called code-blocks, which are entropy encoded according to a bit-plane binary arithmetic coding. Due to its progressive coding, random access capabilities, and the availability of an interactive streaming protocol [14], JPEG2000 is the preferred choice for encoding very high-resolution optical microscopy images that require to be visualized top-to-bottom (i.e., starting from a down-sampled version up to local high-frequency detail) in a responsive way.

H.265/HEVC is a video coding standard [15] which exploits spatial redundancy, through intra-frame prediction techniques, and temporal redundancy enhanced with motion compensation techniques, commonly referred to as inter-frame prediction. Inter-frame prediction is the basis of several encoding modes that reduce redundancy along the third dimension — temporal in video and z-axis in volumetric data — to achieve more competitive compression ratios. The inter-prediction techniques are applied to groups of pictures (GOP) leading to frame inter-dependency, which determines the granularity of the random access to a certain image in the decoding process. Therefore, depending on how fine-grained the random access is required to be, the number of reference images used for predictive coding should be adjusted accordingly (GOP size), as well as the number of images that can be individually decoded (intra-frame period) from the bitstream.

The H.265/HEVC algorithm employs a hierarchical image partitioning scheme that supports blocks from *4* × *4* to *64* × *64* pixels, applying prediction techniques followed by DCT-based transformation as a decorrelation step, and quantization of the coefficients. These are then entropy coded by a context-adaptive binary arithmetic coder, in the last stage of the encoding process. In lossless conditions, the system bypasses transform, quantization, and in-loop filters which are irreversible operations.

Some extensions have been defined to work with the H.265/HEVC core coder, like the Range Extensions (RExt) to make the system compatible with higher image resolutions, bit-depth, as well as alternative color encoding formats. H.265/HEVC also provides scalable modes, which have been adopted by Digital Imaging and Communications in Medicine (DICOM) [16] committee, offering a more efficient way to store and manage medical image data. The targeted applications include using HEVC streams to store and transmit compressed images, but also to produce lossy content out of the archived lossless streams. The H.265/HEVC Scalable Monochrome profile, for example, can represent mono-chromatic sequences with resolution up to 4096 × 2160 pixels, with a bit depth varying from 8 to 16 bits, at 50–60 Hz, in a lossless or lossy format; radiology units working with CT and MRI scanners typically produce such content.

### Related Work

The adoption of lossy over lossless compression schemes in biomedical image representation has long been a controversial topic considering legal obligations that regulate the use of lossless compression for clinical applications due to the potential risk of corrupted data in clinical diagnostics. Notwithstanding, multiple efforts have been made by professional societies in the fields of radiology and pathology to provide objective guidelines for the use of lossy compression in clinical environments. In Europe, the recommendation work [17] proposing the use of lossy compression in radiology units follows other initiatives on the topic [18–20] that suggest maximal compression ratios (CR) ranging from 5:1 to 24:1 for magnetic resonance imaging (MRI) data, and from 5:1 to 12:1 for computed tomography (CT) data. From an assessment by three radiologists on the impact of wavelet-based lossy compression in brain MRI images [21], no statistical differences were reported among the experts for a CR of 20:1, which, in this case, had a distortion of 75 dB. For an increase of the CR to a value of 40:1 (PSNR of 70 dB), only one of the three radiologists demonstrated statistical differences relative to the baseline. More recently, in [22], a compression method based on the encoding of Radon-transform coefficients was proposed, and tested on abdominal MRI images, as well as on axial CT views of the pancreas. In this study, PSNR values from 39 to 50 dB, and SSIM between 0.72 and 0.98 with associated CR between 5:1 and 114:1 were reported, while still preserving the appearance of the images. For the CT scans, PSNR and SSIM values were found to be slightly greater than for the MRI scans (PSNR: 39 to 54 dB and SSIM between 0.79 and 0.98). In [23], a near-lossless compression method based on the factorization of the volume of interest using an optimized multilinear singular value decomposition framework was proposed. In the test dataset (*N* = 12 volumes, including MRI and PET scans), the authors report CR starting at 11:1 up to 37:1, minimum PSNR of 42 dB and minimum SSIM of 0.95, approximately. Another initiative aimed to provide visually lossless image compression was proposed in [24], specifically for dental orthopantomography images. The authors claim that neither artifacts or loss of diagnostically valuable information were induced with the proposed lossy compression solution, which was able to compress the data from 8:1 to 21:1 with a minimum PSNR of 39 dB. Analogously, compression guidelines in the field of clinical pathology have been proposed for optical microscopy. However, different limits of CRs have been reported for compressing microscopy data. In [25, 26], a reference CR of 20:1 was found to generate JPEG 2000 compressed images with indiscernible differences when compared to the original uncompressed content, although CRs up to 75:1 were reported as still appropriate for the diagnosis of *Helicobacter pylori*. The low variability evidenced by the quality metrics used in [27] to evaluate images compressed 8 to 21 times supports the eligibility of lossy image compression for clinical pathology applications. Particularly, PSNR values from 32 to 45 dB (depending on the presence of high-frequency components) were reported in association with CR in the range between 11:1 and 2:1. SSIM was also assessed in this study, and values greater than 0.90 were verified for the same CR range. Additional studies focused on quantifying the impact of lossy compression on the performance of computer aided diagnosis (CAD) tools that are used in routine clinical pathology to support decisions of medical staff. In [28] it was reported that using JPEG 2000 with compression ratios up to a CR of 256:1 did not impact the output of a CAD routine used to segment and characterize the morphology of prostate glands, in the context of prostate carcinoma diagnosis. In another work [29], it was found that after JPEG 2000 compression with compression ratios up to 1:25 and 1:50, the outcomes of automated immunohistochemistry quantification, and breast tumor segmentation were identical.

Most of the works studying the impact of compression on the output of clinical pathology CAD systems were motivated by the increasing data throughput that the field of digital microscopy has experienced. However, and to the best of the authors’ knowledge, similar studies exclusively focusing on non-regulated image data (e.g. preclinical and biological research studies) do not exist, even though the imaging systems responsible for most of the data generated in imaging facilities are exclusively allocated to research studies [30, 31]. Motivated by this and considering the high demand for standardization in preclinical imaging [3], the present study aims to quantify the impact of standard lossy image compression in the context of a multimodal preclinical study involving image data generated by radiology and optical imaging systems. The study considers as target data compression methods of the state-of-the-art H.265/HEVC and JPEG 2000 ISO standard encoders, widely used in commercial equipment and with robust and open-source implementations.

## Materials and Methods

### Imaging and Image Analysis Protocols

#### Imaging & Sample Preparation Protocols

Image acquisition protocols and sample preparation for all modalities were detailed in [11]. Below, we summarize the acquisition and sample preparation procedures for µMRI, µCT, and HREM based on [11], and Table 1 summarizes the representation, spatial resolution, and file size of a single tomographic imaging stack for the three modalities.

**Table 1:** Main characteristics (representation, spatial resolution, and size) of the multimodal test sequences used in the present study

**Modality**	**Bit depth**	***X* (px)**	***Y*(px)**	***Z* (slices)**	**Resolution (µm)**	**Raw file size (MB)**
µ-CT	16	579	355	681	34 × 34x34	300
HREM	16	2150	2500	4730	3 × 3x3	50 800
µ-MRI	16	440	466	92	60 × 60x200	36

Once murine tumors reached a size of 20–70 mm3, µMRI was conducted using a 15.2 T Bruker system (Bruker BioSpec, Ettlingen Germany), and a 35-mm quadrature birdcage coil. To visualize blood vessels, a 3D fast imaging technique was employed, utilizing a steady state free precession (FISP) sequence with a gradient echo readout. The imaging parameters were as follows: repetition time (TR)/echo time (TE) = 5.7/2.85 ms, 30° flip angle, 30 × 30 × 10 mm3 field of view, 500 × 500 × 50 matrix size, 60 × 60 μm^2^ in-plane resolution, 200-μm slice thickness, and number of experiments [NEX] = 16. The resolution of the obtained images (refer to Table 1) was calculated based on the field of view divided by the number of frequency and phase encoding steps (i.e., the imaging matrix). During image acquisition, mice were anaesthetized using isoflurane (4% induction, maintained at 1.5%).

For µCT (SCANCO microCT 50, SCANCO Medical AG, Brüttisellen, Switzerland), the whole animal was perfused with a contrast agent (30% Micropaque), which is a mixture of barium sulfate and 2% procine gelatin, immediately postmortem. The perfusion surgery was performed as described in [32] prior to application of the contrast agent. For fixation, the animal was perfused with saline containing 1,000 IU heparin, followed by a perfusion of 4% formaldehyde, and another perfusion of saline. Each perfusion step took approximately 5 min and was done manually using a syringe. After perfusion fixation, the contrast agent mixture, heated to 40–50 °C, was injected into the animal using a 20-ml syringe. Once the femoral artery and adjacent vein were filled with the contrast agent, the perfusion was complete, and the animal was stored at 4 °C for 24 h-to allow the contrast agent to cure. The tumor and surrounding tissues were then extracted and placed in a 15-ml centrifuge tube. The extracted tissues were scanned using a SCANCO microCT 50 at 90 kVp, 200 µA with 1000 projections per 180° integrated for 400 ms, with a 20.48 mm field of view, and reconstructed to an isotropic voxel size of 10 µm. The scanning time for each sample varied between 105 and 145 min depending on the tumor size. The resolution of the reconstructed scans depended on the scanning parameters, such as the field of view and binning, and was chosen to balance the scanning time and sample size (see Table 1).

HREM is an optical block face imaging technique that generates 3D volume data by aligning a series of digital images. This ex vivo method involves histologically processing specimens and embedding them in methacrylate resin blocks dyed with eosin. The blocks are then physically sectioned using a microtome or microtome-like device, while a microscope equipped with fluorescent filters and a basic digital camera captures subsequent images directly from the block surface during the sectioning process [2]. For HREM, tumor samples were dissected and subjected to a series of processing steps. First, the samples were washed in PBS for a duration of 2 days, followed by dehydration using ethanol of increasing concentrations. Specifically, the samples were soaked in 30%, 50%, and 70% ethanol for 24 h each, 80% ethanol for 16 h, 90% ethanol for 3 h, and 100% ethanol (with two changes) for 6 h. The 70%, 80%, 90%, and 100% ethanol solutions contained 0.4 g of eosin per 100 ml, which was sourced from Waldeck GmbH & Co. KG in Germany. After dehydration, the samples were treated with JB-4 solution A, which contained 1.25 g of catalyst (benzoyl peroxide, plasticized) and 0.4 g of eosin per 100 ml, and infiltrated for 7 days (with three changes) at 4 °C. The samples were embedded in eosin-dyed JB-4 using a standard protocol and oriented with the proximal end beneath the future block surface. The molds were sealed airproof and allowed to polymerize for 2 days. After baking the polymerized blocks for 48 h at 80 °C, they were stored at room temperature until HREM data generation. HREM volumes of interest comprising approximately 5000 single digital images were generated in a fully automated way in about 9 h. The data were acquired using a custom-made instrument according to a standard protocol as outlined in [33]. The pixel dimensions of each section were 2.96 × 2.96 µm2, with a section thickness of 3 µm.

#### Segmentation Protocols & Datasets

For µMRI, a maximum intensity projection was used to visualize the vasculature in 3D. Raw data were acquired and exported into AMIRA [34] as NIfTI files. For µCT, reconstructions were performed directly with the SCANCO microCT software and stored as ISQ files. For further processing, the scan was exported as DICOM stacks. For HREM, image datasets were saved and processed as tiff files, using volume rendering to visualize the tumor and tissues. Segmentation and visualization were carried out using the software Amira 2020.1 (Thermo Fisher Scientific). For HREM, because of unspecific tissue contrast, manual tracing of vessel outlines in image sections was necessary and, due to the huge HREM files’ size of up to 50 GB, the workloads would surpass the feasibility of this study. Therefore, a volumetric region of interest was selected by another experienced researcher and used on the subsequent steps aimed to quantify the impact of lossy compression. The segmented results were exported as binary images to generate blood vessel masks.

The segmentation started with the original datasets of the three modalities by a single researcher (Segmentor #1). Afterwards, the compressed data sets were segmented in random order concerning the compression degree. After a resting period of several days, the initial segmentation of each data set was re-examined searching for errors and missing parts. There was an attempt to spend the same amount of time segmenting each image stack, both original and compressed ones. To compare the segmentation accuracy in a compression-free scenario (inter-observer variability), another researcher segmented the original HREM and MRI datasets (Segmentor #2). In addition, each dataset was reassessed after compression by Segmentor #1 and compared to his previous results, to quantify the intra-observer variability.

### Configuration of the Image Encoders

JPEG 2000 and H.265/HEVC offer distinct mechanisms to control the distortion induced by the compression since the underlying rate-control mechanisms are based on different concepts. On one hand, JPEG 2000 allows to specify a target Peak Signal-to-Noise Ratio (PSNR) that affects the number of bits per pixel in the compressed image allocated to represent the encoded images at each coding pass of the entropy coding step. Higher PSNR values correspond to less distortion, i.e., less compression-related image quality degradation. On the other hand, H.265/HEVC makes use of a quality-control mechanism according to the so-called quantization parameter (QP). QP determines the scale of the discrete levels used to quantize the coefficients obtained after transforming the prediction residuals to the spatial frequency domain using a discrete cosine transform (DCT). Hence, high QP values correspond to a rough quantization, which makes the preservation of fine, and high-frequency details more difficult after compression, but results in less bits per pixel used to represent the (compressed) image data.

To obtain comparable compressed files using both encoders, a constrained range of objective quality (PSNR) values, between 40 and 60 dB, was set as the target quality for the JPEG2000 encoder, given that this range of PSNR values is associated to a visually lossless compression regime (considering natural image content). The configuration of the JPEG2000 compression corresponds to the one implemented by the default call to the OPENJPEG executable, with the target PSNR passed as an additional parameter.

Once all the slices were encoded, the volumetric sequence was reconstructed and the global PSNR was computed against the respective original/raw sequence. The encoding of the same data using H.265/HEVC involved the use of a heuristic procedure to determine the values for the QP parameter guaranteed that the resulting H.265/HEVC compressed files achieved similar objective quality (PSNR) to the ones obtained by JPEG 2000 compression. The HEVC/HEVC-RA + RExt profile was configured with an Intra-Frame period and GOP size of 8, maximum coding unit size of 64 × 64, and with the fast search method for motion estimation.

### Task-Oriented Objective Assessment

**Fig. 1:**
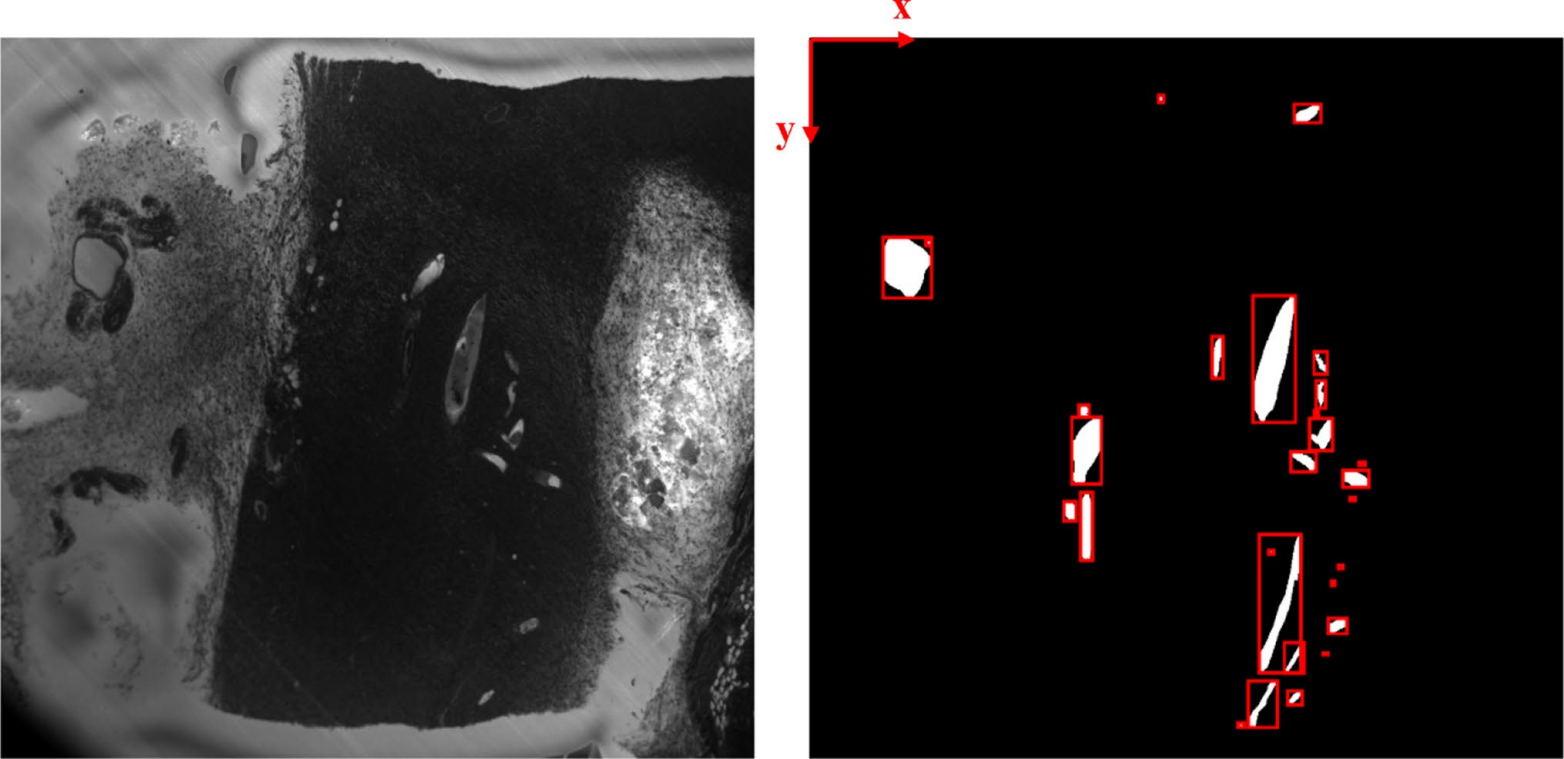
Example of a raw HREM slice (left) with the corresponding annotations (right) enclosed by red bounding boxes

Based on the published CMI feasibility study to visualize murine tumor vasculature across scales [11], we compared the morphological preservation of blood vessels within regions of interest after lossy compression. The blood vessel morphology was extracted and quantified using manual and automated segmentation. The reference data corresponded to the manually segmented blood vessels of the raw datasets. The MRI (Fig. 1) and HREM (Fig. 5) sequences were used to quantify the impact of lossy compression on the performance of a manual segmentation task, whereas the CT sequence (Fig. 3) was used to quantify the impact of compression on the performance of an automated approach using Otsu’s method for automatic thresholding [35]. For the HREM and MRI image sequences, the available segmentation masks included:

- the reference mask obtained after manual segmentation of the raw image data (annotations of Segmentors #1 and #2);
- a second reference mask drawn manually on the raw image data with a time lapse of 1 month to the first reference annotation (obtained by Segmentor #1 only);
- the masks drawn on each decompressed version, referred to as *compressed masks* (obtained by segmentator #1 only).

The masks in a) and b) were used to compute the inter- and intra-variabilities according to the method described in the “Intra-observer Variability” and “Inter-observer Variability” sections). For the CT image sequence, the pool of available segmentation masks included:

- the reference mask obtained after automated segmentation of the raw/uncompressed image data;
- the masks obtained after automated segmentation of each decompressed version.

Based on the available data, an *N-to-1* format was used to compare the N compressed masks against the corresponding reference mask according to two objective scores typically employed in biomedical image segmentation problems [36]: Jaccard similarity coefficient, or simply Jaccard Index (JI), and the average Hausdorff Distance (HD). The JI consists of a widely adopted overlap measure in segmentation problems. Given two binary images X and Y, it is determined by dividing the number of annotated pixels at coincident positions by the total number of annotated pixels, according to the expression:

1 $$JI\left(X,Y\right)=\frac{x \cap y}{x \cup y}$$

which is also referred to as “Intersection over Union” (IoU). In this study, the JI was computed per slice and arithmetically averaged for the entire 3D stack.

In addition, the average HD was also included in this study since it has also been reported as an appropriate metric for medical image segmentation problems [37], particularly in the presence of complex, and thin structures [38]. In order to compute the HD between a pair of binary volumes X and Y, their corresponding sets of annotated points, herein represented by the discrete sets $\left\{X\right\}$ and $\left\{Y\right\}$, have to be extracted. When computing the HD between X and Y, the set of directed Euclidean distances from X to Y ${d}_{XY}$, and its reciprocal set of distances ${d}_{YX}$ are required to be known. ${d}_{XY}$ can be obtained by iterating along $\left\{X\right\}$ and finding the closest element in Y to the point ${x}_{i}$, as given by the expression below.

2 $$\left\{d_{XY}\right\}=\left\{min\;d\left(x_i,Y\right)\right\},\;x_i\in\;X.$$

After computing the reciprocal set of distances $\left\{{d}_{YX}\right\}$, the average HD between point sets X and Y is defined as

3 $$HD\left(X,Y\right)=\frac{\left(\frac{1}{\parallel X\parallel }\sum {d}_{XY}+\frac{1}{\parallel Y\parallel }\sum {d}_{YX}\right)}{2}$$

with ||X|| and ||Y|| representing the number of points in distributions X and Y, respectively. To assess the inherent variability of the manual segmentation process (i.e., to determine and normalize the best achievable JI and HD values), the intra-observer variability (IaOV) and the inter-observer variability (IeOV) was calculated as described in 2.3.1 and 2.3.2.

#### Intra-observer Variability

As mentioned, the observer responsible for annotating the structures of interest on the compressed files (Segmentor #1) segmented the raw sequence twice with a time interval of approximately one month (${S}_{t=0}$ and ${S}_{t=1}$, respectively). This allowed us to perform the reference pairing $RP:\left({S}_{t=0},{S}_{t=1}\right)$ to characterize the reproducibility of its annotations in a compression-free scenario. For this, the set of slices of the raw dataset was identified, in which the observer performed coherently between time-points ${t}_{0}$ and ${t}_{1}$. Since the main goal of this study aims to interpret the quantitative impact of lossy image compression on the segmentation outcome, this step was crucial because otherwise the variation of the segmentation could have been mistakenly attributed to the compression. An empirically selected threshold $t: JI\ge 0.7$ was used to filter out the slices with coherent segmentation $V$ from the $\Vert N\Vert$ available slices. Here, $V$ represents the set of slices where the intra-observer agreement in a compression-free scenario was verified according to

4 $$V:{\left\{i,if\;JI\left(S_{t=0,i},S_{t=1,i}\right)\geq t\right\}}_{i\in\parallel N\parallel}$$

The final IaOV value corresponds to the average of the JI between $\left({S}_{t=0},{S}_{t=1}\right)$, computed only at slices $i\in V$, as given in Eq. 5

5 $$IaOV\left(JI\right)=\frac{{\sum }_{i}^{V}JI\left({S}_{t=0;i},{S}_{t=1;i}\right)}{\Vert V\Vert }$$

A similar approach was followed to compute a IaOV figure based on the HD similarity metric. Specifically, when converting the reference binary volumes into discrete sets of annotated points, all the annotated voxels defined in slices that do not belong to a valid set $V$ were ignored.

#### Inter-observer Variability

A second observer segments the raw HREM and MRI sequences following the same protocol as the first observer, and the segmentation data was used to compute an inter-observer variability (IeOV), according to the following computational methodology. Starting from the sets of coherently segmented slices specific to each observer (A and B), ${V}_{A}$ and ${V}_{B}$, the set of slices where both observers achieved high agreement between their first and second segmentation on the raw data was represented by the logical conjunction ${V}_{{O}_{AB}}={V}_{{O}_{A}}\cap {V}_{{O}_{B}}$. ${V}_{{O}_{AB}}$ hence represents the set of slices where the segmentations of both observers are compared to define the IeOV. Since each observer created two reference masks for each modality, the next step involved merging these two segmentations $\left({S}_{t=0},{S}_{t=1}\right)$ into one. The merging operation consisted of a logical intersection in the form of ${S}_{t=0}\cap {S}_{t=1}$ to keep only the annotations that are preserved between ${t}_{0}$ and ${t}_{1}$ time-points. Once the pair $\left({S}_{t=0},{S}_{t=1}\right)$ of each observer was merged, the two resulting binary masks ${S}_{A}$ and ${S}_{B}$ were used to compute the JI for the slices belonging to ${V}_{{O}_{AB}}$. The inter observer variability, IeOV, is then defined as the average of the JI values of the slices in ${V}_{{O}_{AB}}$, as given by Eq. 6

6 $$IeOV\left(JI\right)=\frac{{\sum }_{i}^{{V}_{{O}_{AB}}}JI\left({S}_{A;i},{S}_{B;i}\right)}{\Vert {V}_{{O}_{AB}}\Vert }$$

### Texture Analysis

Due to the lack of prior results in the scientific literature, it is not clear how the compression affects the image texture of “biomedically relevant” regions, and how the compression effects vary with the compression ratio. To ensure that the varying levels of compression induced real variations on the image statistics at those regions, a prior quantitative texture analysis was accomplished before sharing the compressed data with the expert observers. First, the reference segmentations were used to find the isolated annotations/segments at each slice. By iterating over all the annotations ${A}_{n=1,..., N}$, each one was represented by the respective bounding box ${B}_{n}$ (see Fig. 1).

At each bounding box, a weighted version of the isotropic total variation operator $wTV{B}_{n}$ given by was computed. In this equation, $y{Top}_{{A}_{n}}$ and $x{Left}_{{A}_{n}}$ refer to the top y-coordinate and left x-coordinate, respectively, of the bounding box enclosing the ${A}_{n}$ annotation. H and W correspond to the height and width of each bounding box ${B}_{n}$, respectively, and I represents the image intensity.

7 $$wT{V}_{{B}_{n}}=\frac{{\sum }_{i=yTo{p}_{{A}_{n}}}^{yTo{p}_{{A}_{n}}+H-1}{\sum }_{i=xLef{t}_{{A}_{n}}}^{xLef{t}_{{A}_{n}}+W-1} \sqrt{{\left|I\left(i+1,j\right)-I\left(i,j\right)\right|}^{2}+{\left|I\left(i,j+1\right)-I\left(i,j\right)\right|}^{2}}}{H\times W}$$

The total variation operator leads to a measure of image complexity with respect to the spatial variation of the image texture, and since each bounding box includes not only the annotation of the object but also part of the background, the value of the total variation operator indicates the ability to delineate/segment the object. Particularly, the descriptor defined in Eq. 7 is more sensitive to regions where the image intensity varies with greater magnitude, which are particularly important for manual segmentation tasks. Once all the bounding boxes were processed, the distribution of $wTV$ values was averaged, and the resulting value represented the texture sharpness of each compressed image (Table 2). The values obtained demonstrate that the texture complexity at relevant regions decreases with the increase of the compression level, and in general, the magnitude of this decrease is greater using JPEG 2000 compression.

**Table 2:** The impact of compression ratio on the image details, grouped by modality and encoder. The values represent the percentage variation of the average weighted total variation (wTV) of each compressed file relatively to the raw data

**CT**				**HREM**				**MRI**			
**HEVC**		**JPEG2000**		**HEVC**		**JPEG2000**		**HEVC**		**JPEG2000**	
**CR**	$\Delta wTV (\%)$	**CR**	$\Delta wTV (\%)$	**CR**	$\Delta wTV (\%)$	**CR**	$\Delta wTV (\%)$	**CR**	$\Delta wTV (\%)$	**CR**	$\Delta wTV (\%)$
**1**	0	**1**	0	**1**	0	**1**	0	**1**	0	**1**	0
**16**	−1.2	**8**	−0.6	**45**	−1.6	**10**	−0.1	**10**	−2.2	**9**	−7.3
**34**	−1.9	**9**	−1.2	**112**	−3.0	**33**	−1.7	**14**	−4.0	**15**	−26.7
**58**	−4.0	**12**	−25.6	**274**	−6.3	**159**	−11.4	**34**	−7.7	**44**	−55.9
				**941**	−16.4	**643**	−47.7	**52**	−10.0		

## Results

The present section includes the results of the segmentation-assessment, generated according to the setup detailed in the “Materials and Methods” section for each encoder and imaging modality. The test datasets corresponds to the compressed files obtained as described in the “Configuration of the Image Encoders” section, and Table 3 starts this section to summarize the achieved compression ratio of all the compressed files, and the target quality metric used by the encoders.

**Table 3:** Compression ratios and objective target quality for each encoder and modality combination

**HREM**					
**Encoder**	**Target quality**	**Compression ratio**	**Encoder**	**Target quality**	**Compression ratio**
JPEG 2000	65 dB	10	H.265/HEVC	60 dB (QP 2)	45
	60 dB	33		58 dB (QP 5)	112
	55 dB	159		56 dB (QP 10)	274
	50 dB	643		54 dB (QP 17)	941
**µMRI**					
JPEG 2000	55 dB	9	H.265/HEVC	57 dB (QP 2)	10
	50 dB	15		54 dB (QP 5)	14
	45 dB	44		49 dB (QP 10)	34
				48 dB (QP 12)	52
**µCT**					
JPEG 2000	57 dB	8	H.265/HEVC	53 dB (QP 5)	16
	55 dB	9		50 dB (QP 8)	34
	53 dB	12		48 dB (QP 10)	58

### Qualitative Assessment of the Compression Schemes and Preservation of Vascular Structures

**Fig. 2:**
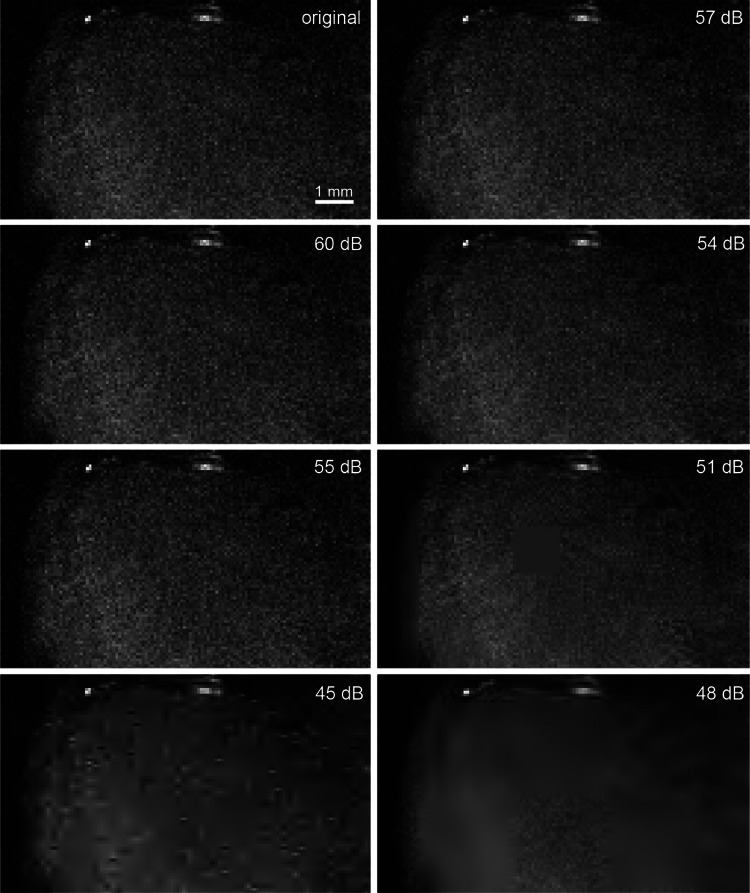
µMRI of a mouse tumor after compression. The same region cropped from a slice of the MRI stack is displayed at different qualities (left column – JPEG 2000; right column – H.265/HEVC). Note the H.265/HEVC blocking artefacts starting for 51 dB image quality. Note the high quality of the information preserved in images compressed with higher qualities, and all the JPEG 2000 compressed versions up to a target quality of 55 dB

**Fig. 3:**
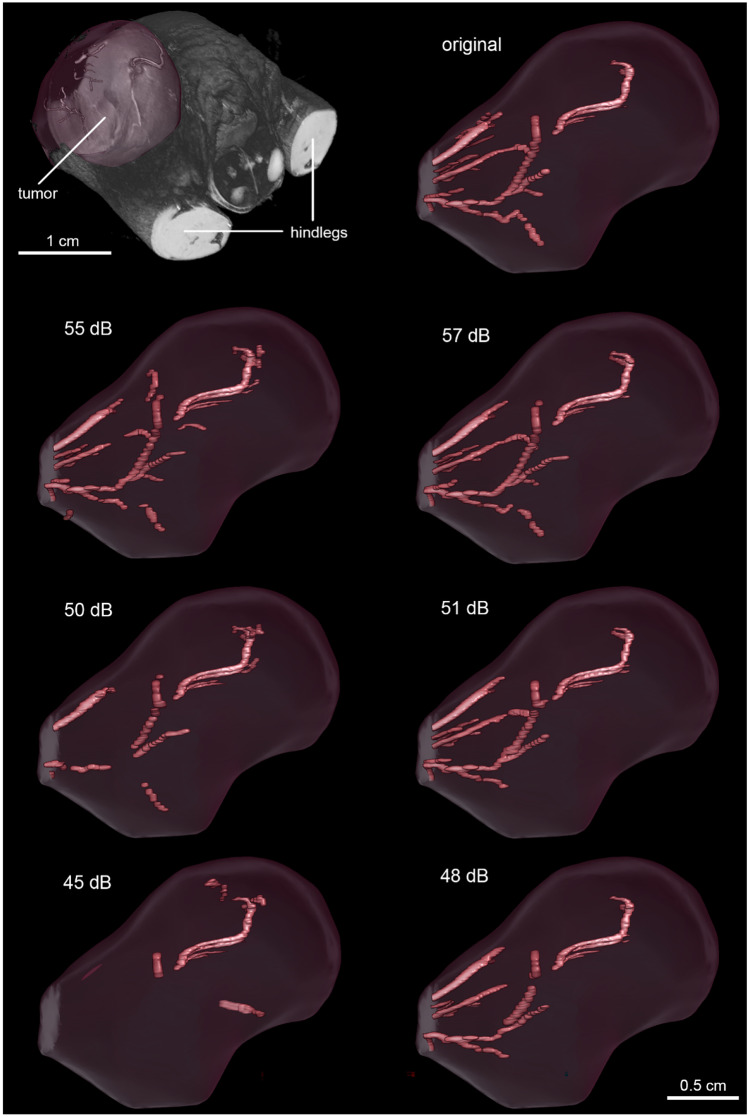
Segmented blood vessels in a mouse tumor extracted from images with varying level of compression (left column – JPEG 2000; right column – H.265/HEVC). The location of the mouse tumor sample imaged with µMRI and illustrated via a volume rendering combined with a surface rendering on the top left. The tumor is situated on the dorsal side of the left hip. In the images beneath, the tumor is shown separately, using surface and volume rendering. As compression increases from top to bottom fewer vessels are visible inside the tissue. Particularly, the image compressed with JPEG2000 with a target PSNR of 45 dB stands out, as many vessels are missing

For µMRI and HREM, only vessels inside the tumor tissue were segmented. The image quality decreased with increasing compression ratios for both compression schemes JPEG 2000 and H.265/HEVC. Different compression artefacts occured, which made the segmentation process and the visual vessel identification by the expert more difficult and error prone. For example, the µMRI JPEG 2000 compressed dataset with target quality of 45 dB (Fig. 2) demonstrates strong artefacts in a cross-like pattern. Upon visual inspection by Segmentors #1 and #2, it was found that, below 55 dB blood vessels were not faithfully identified anymore, and the position of the vessels becomes uncertain (Fig. 3). Despite its coarse and blurry appearance, the slice compressed with H.265/HEVC with a target QP of 12 still preserved the big vessels and their morphology (bright spots at the top).

**Fig. 4:**
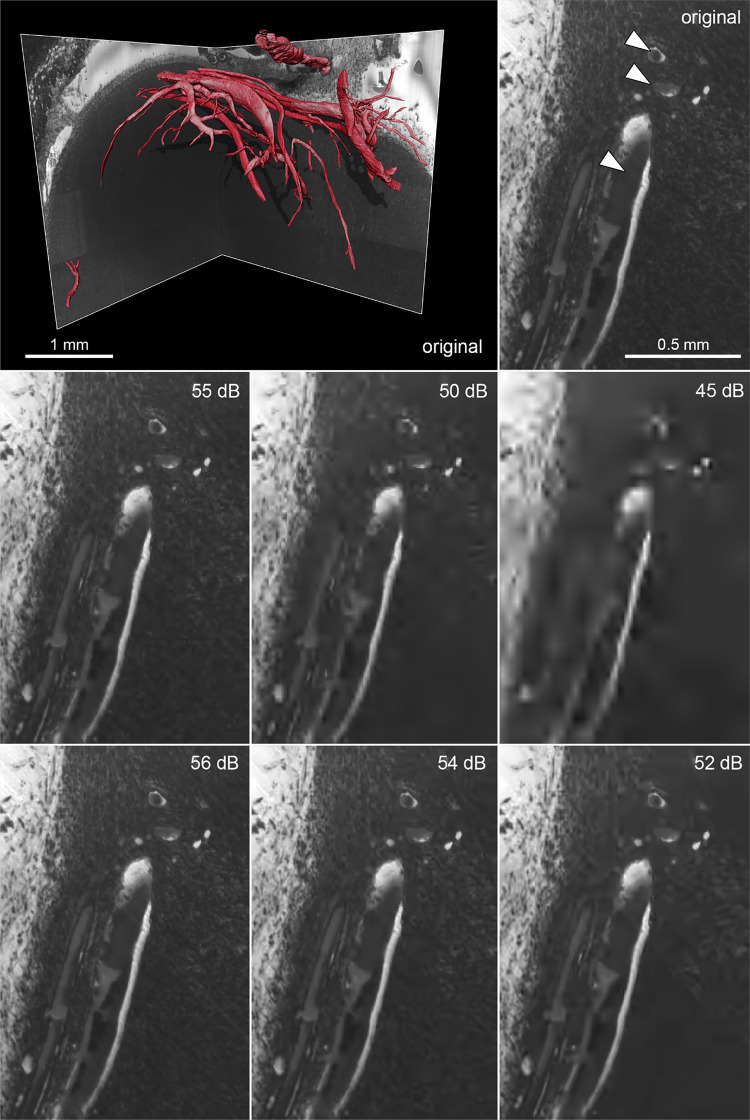
HREM images of a mouse tumor region extracted from images with varying level of compression. Left top corner: HREM image analysis, displayed via a volume rendering in combination with the image planes y,z. Other: Images of the same slice compressed with different quality (mid-row: JPEG 2000 compression; bottom row: H.265/HEVC compression). The top right corner shows the original image, triangles mark segmented vessels. The highest H265/HVEC compression (bottom-right corner) resulted in detailed images in contrast to the highest JPEG2000 compression (target quality of 45 dB), which shows artefacts and a blurry appearance

**Fig. 5:**
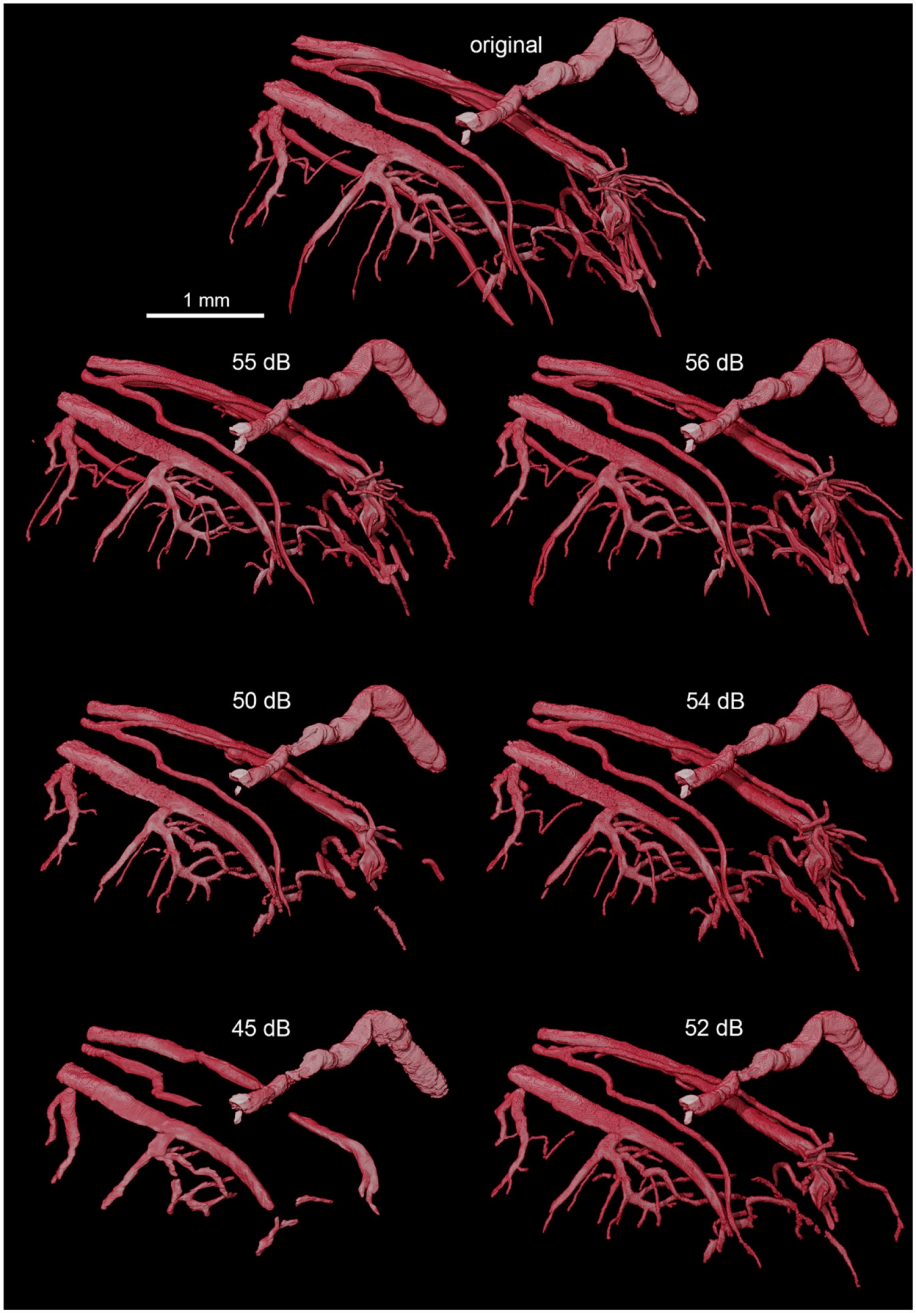
Volume rendering of the segmented vessels of a mouse tumor HREM image stack after compression with varying levels (left column – JPEG 2000; right column – H.265/HEVC). Note the decrease in number and area of the segmented vessels using JPEG 2000 with a target quality of 45 dB. From 55 to 50 dB, JPEG 2000 shows differences mainly at the level of microvessels (capillaries)

Regarding HREM, as for the visual inspection of µMRI datasets, the JPEG2000 compression method seems to preserve less vascular structures than the HEVC compression. Using a target PSNR of 55 dB with JPEG 2000, some minor artefacts and blurriness can be observed (Fig. 4). However, below a target PSNR of 50 dB, major visual artefacts are created, which in turn result in incomplete morphology identification (Fig. 5) where a reduction of the vessels’ length, as well as the vanishing of other, typically smaller vessels, is visible. A very significant reduction of the volume of the segmented vasculature network can be verified using JPEG 2000 with a target PSNR of 45 dB. This degradation, however, is not so evident with the increase of H.265/HEVC compression, which yields better results comparatively to JPEG 2000. In fact, for H.265/HEVC, although higher compression still leads to missing and shorter vessels, the overall morphology resembles more the one visible in the original file.

**Fig. 6:**
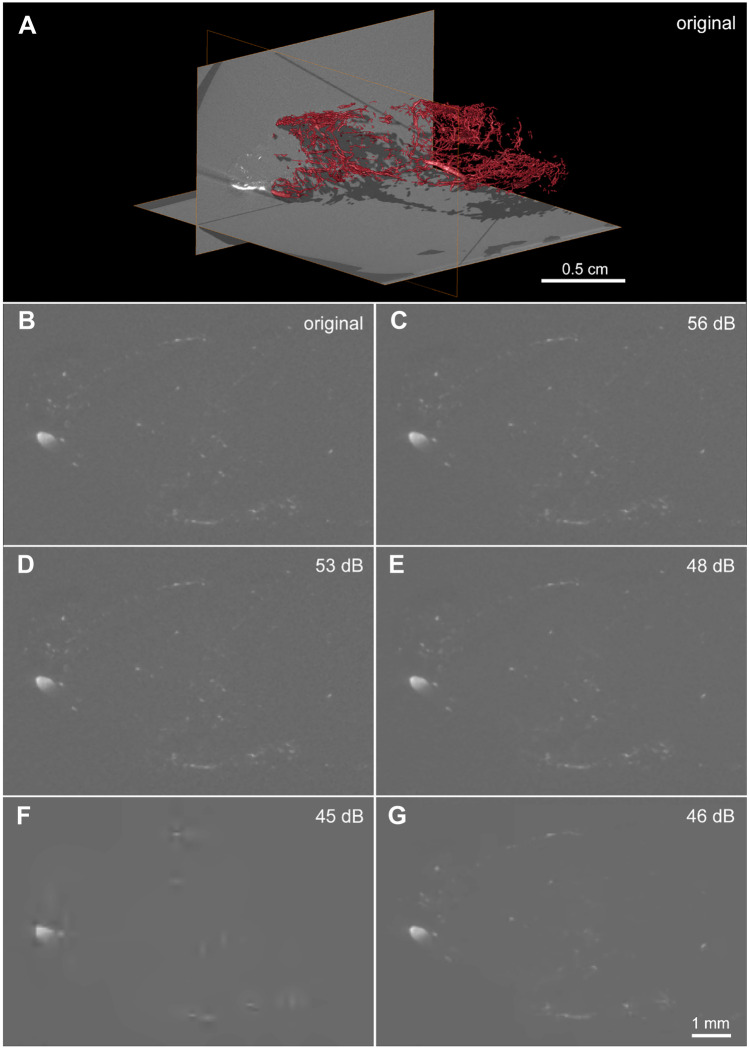
µCT images of a mouse tumor vasculature extracted from an image with varying level of compression (left column – JPEG 2000; right column – H.265/HEVC). Visualization of the original data with volume rendering on the top (**A**). **B**–**G** One slice of the CT stack, sampled from different compressed files. Note the blurred structures in **F** and **G**, notably affected by aggressive low-pass filtering characteristic of the lossy compression algorithm

**Fig. 7:**
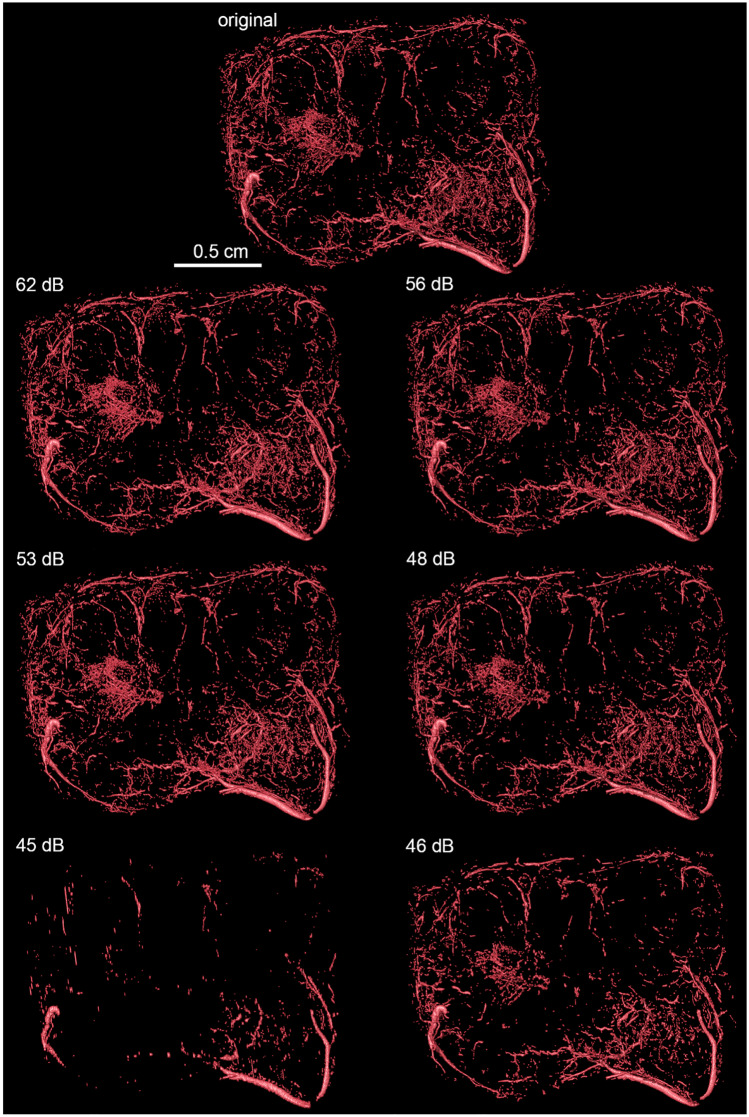
Comparison of the vasculature depicted in Fig. 6, imaged by µCT, and displayed via volume renderings compression (left column – JPEG 2000; right column – H.265/HEVC). Note that only slight differences appear in JPEG 2000 (62 dB and 53 dB), and H.265/HEVC (56 dB) in comparison to the original. Changes of the segmentation are more visible for H.265/HEVC 48 and 46 dB, but especially using JPEG 2000 with target quality of 45 dB, where only the biggest vessels are segmented properly

For µCT, in contrast to both other modalities, the segmentation was carried out automatically. Figures 6 and 7 show that changing the compression parameters leads to changes on the segmented vasculature. For both H.265/HEVC and JPEG 2000 a decrease in volume, length and number of vessels was observed for all compressed versions. The volumes compressed with H.265/HEVC showed only slight visual differences of the vasculature up until the target QP of 8, where the images become less noisy, compared to the original (Fig. 6). From a target quality of QP10 to QP20 a decrease in the vessel length can be observed. The JPEG2000 compression differs, as the image stack becomes unreliable in dB45. In dB40, the information of any vessels inside the stack is lost (therefore not shown).

**Fig. 8:**
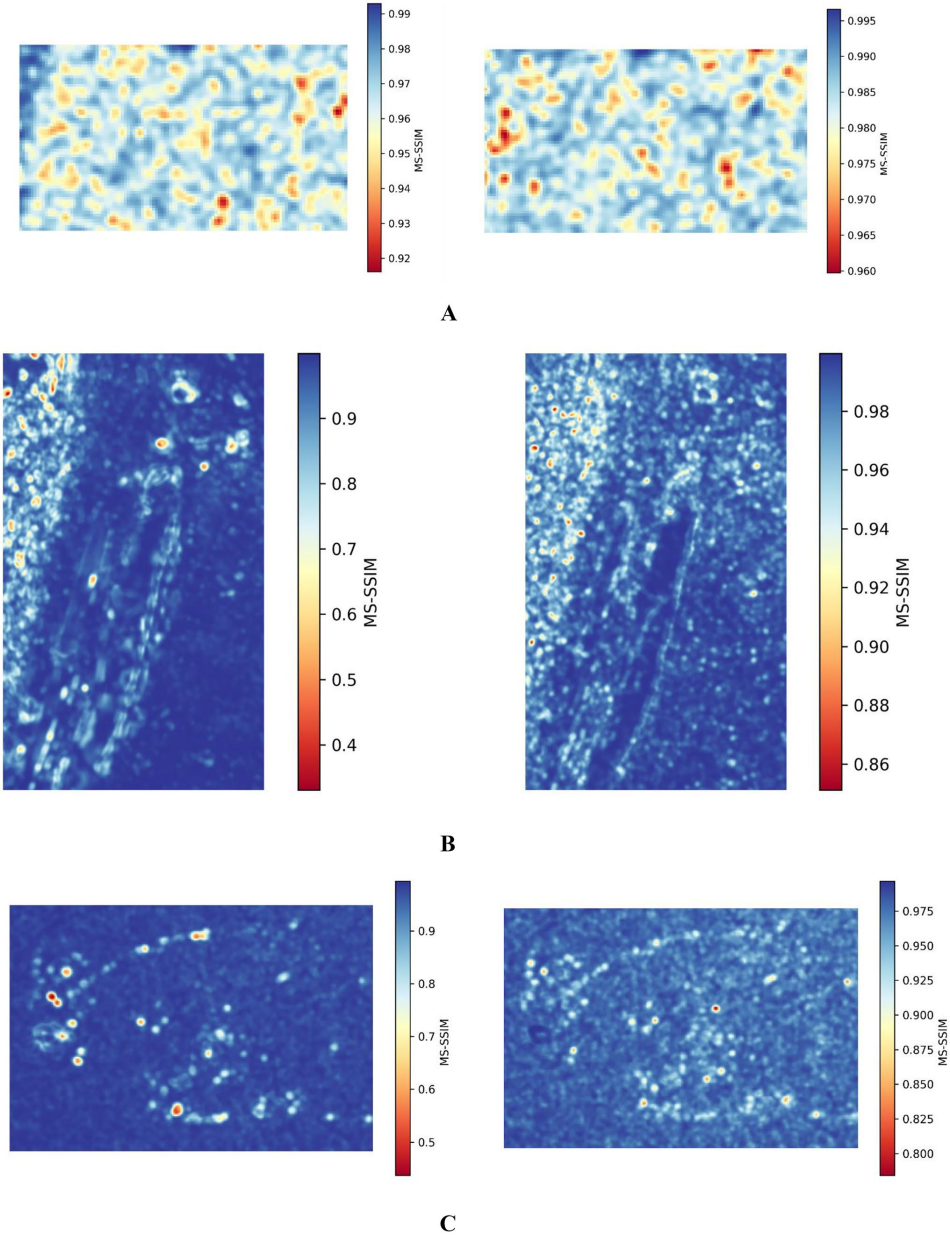
MS-SSIM maps between compressed and uncompressed images. Images A, B, C correspond to regions depicted in Figs. 2, 4, and 6, respectively. Left column: MS-SSIM between the raw image and the corresponding most compressed JPEG 2000 image. Right column: MS-SSIM between the raw image and the corresponding most compressed H.265/HEVC image

Overall, for all three modalities, and compared to JPEG2000, H.265/HEVC compressed images resulted in better manual segmentation, and improved preservation of vascular parameters such as vessel length and morphology at similar CRs (Fig. 7). Figure 8 provides a compact view of the multi-scale structural similarity index measure maps (MS-SSIM) [39], computed at regions depicted in Figs. 2, 4, and 6 for the highest compression tested for both JPEG2000 and H.265/HEVC. First of all, the most compressed H.265/HEVC images presented slightly better quality than their JPEG2000 homologous (HREM: H.265/HEVC – 52 dB, JPEG2000 – 45 dB; CT: H.265/HEVC – 46 dB, JPEG2000 – 45 dB; MRI: H.265/HEVC – 48 dB, JPEG2000 – 45 dB) and, as expected, the MS-SSIM maps also evidence an overall improved performance of H.265/HEVC in retaining structural detail in comparison to JPEG2000. Furthermore, the MS-SSIM maps computed at JPEG2000 compressed images demonstrate a greater dispersion, denoted by greater difference in terms of structural detail between background (less complex) regions and foreground regions. MS-SSIM maps of H.265/HEVC compressed regions, on the other hand, show less dispersion with a more homogenous distribution of structural detail preservation.

### Quantitative Assessment

**Fig. 9:**
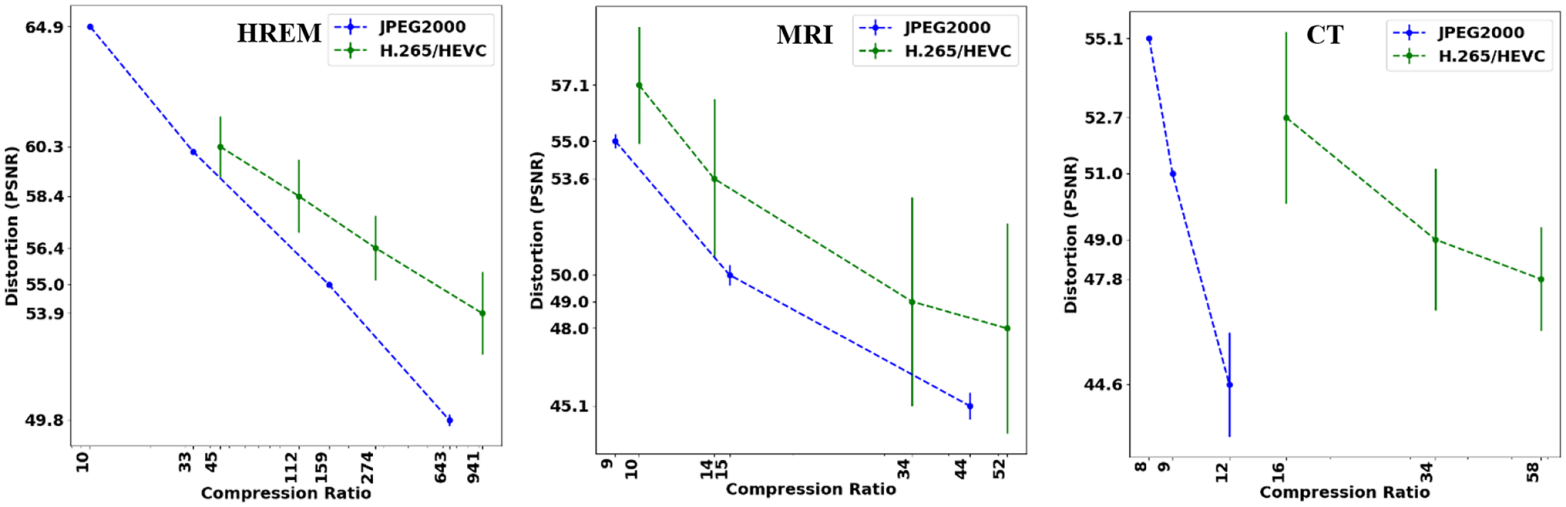
CR-quality curves for each modality, including H.265/HEVC compressed files (green lines) and JPEG 2000 compressed files (blue lines)

The quantitative assessment of the compressed image fileswas characterized by two metrics: compression ratio (CR) and image quality. A CR-quality curve relates the distortion magnitude introduced by the compression with the number of bits required by the encoder to represent the image. Figure 9 depicts the performance of both encoders, for each modality, through graphical representations of the correspondence between compression ratio and the corresponding image quality value (in this case PSNR). The range of distortion values after compression with JPEG2000 and H.265/HEVC encoders was found to be between 45 and 65 dB. Furthermore, the curves highlight the superior compression efficiency of H.265/HEVC over JPEG 2000 and, as expected lower quality associated with larger compression ratios. For the µCT sequence, the compression ratios for H.265/HEVC and JPEG 2000 did not match, since, from a compression ratio of approximately 12 onwards, the JPEG 2000 compressed versions did not reach the minimum quality required for automated processing, as reported independently by the two observers. This can be due to the very thin vascular structures that are difficult to represent in an efficient way using a DWT-based coding scheme. In line with the qualitative assessment, the quantitative evaluation of the compressed files according to the approach described in the “Texture Analysis” section, showed a gradual degradation of the image sharpness at regions corresponding to annotated vessels.

**Fig. 10:**
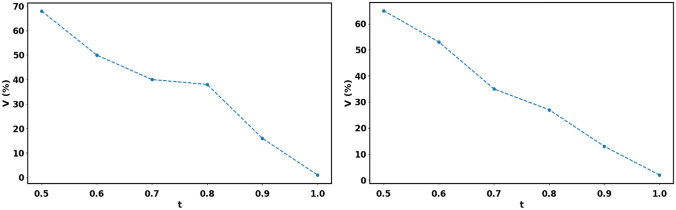
Percentage of the total number of slices kept in the volume after applying the overlap threshold between the first and second reference segmentations on raw HREM (left) and MRI (right) sequences

**Fig. 11:**
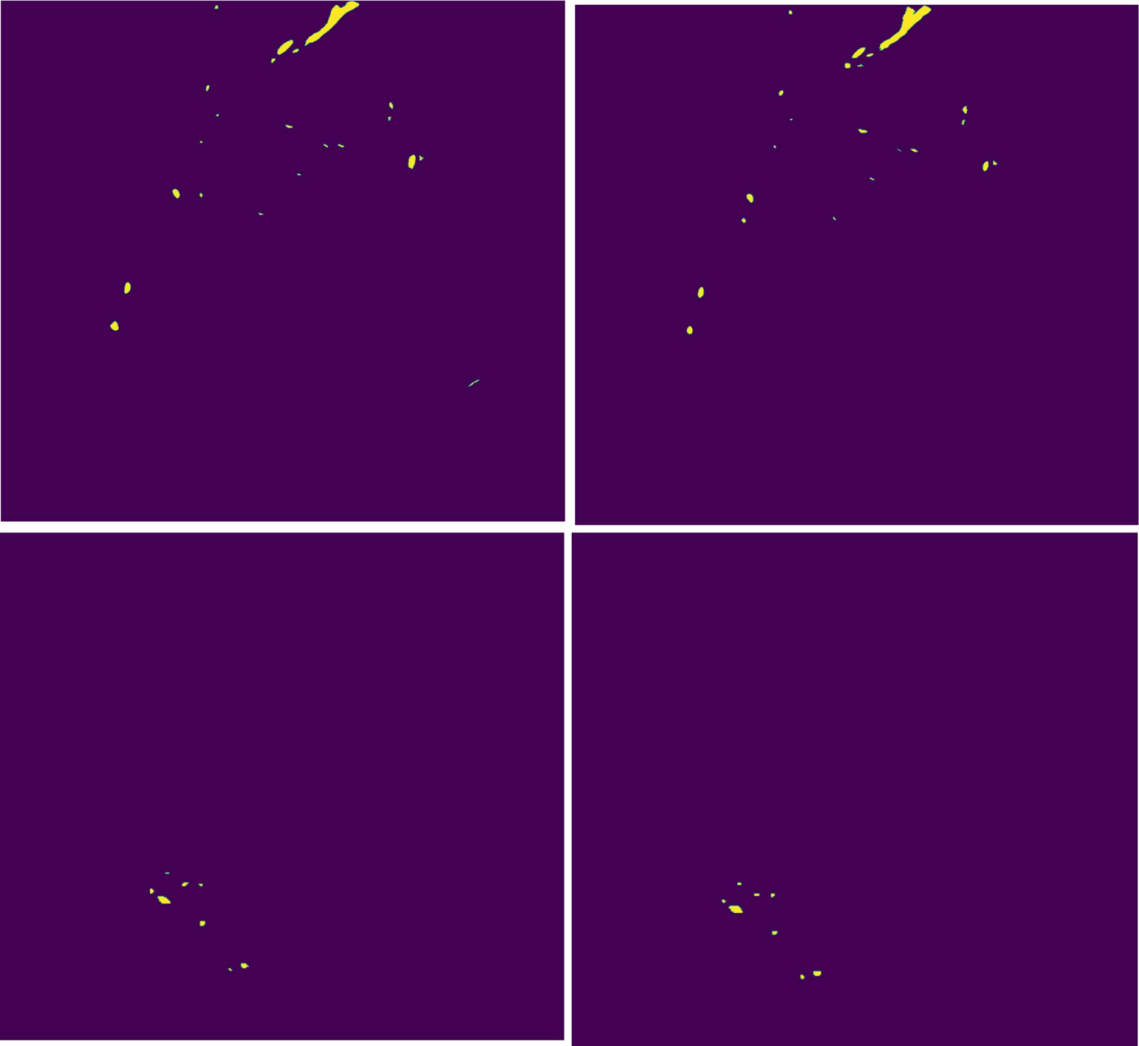
Two pairs of slices (top row: HREM, bottom row: MRI) extracted from the first (t = 0; left image) reference annotation, and on the right from the second (*t* = 1) reference annotation. The JI between the HREM annotations (top row) is 0.70, and the JI between the MRI annotations (bottom row) is 0.73

To quantitatively compare the compressed and the original datasets across modalities, only non-compressed slices where the intra-observer variability score (i.e., the Jaccard Index) was above a minimum acceptable value *t* were chosen. Figure 10 shows how changes in the parameter $t$ determine the size $\Vert V\Vert$ of the set of working/valid slices of HREM and MRI sequences. After visual inspection of the two HREM and MRI control segmentations, the threshold was set to $t=0.7$, which led to retaining more than one-third of the original number of slices for both modalities. It can be visually analyzed in Fig. 11 that a good preservation of the structural detail is still obtained with $t=0.7$, as suggested in [40].

**Fig. 12:**
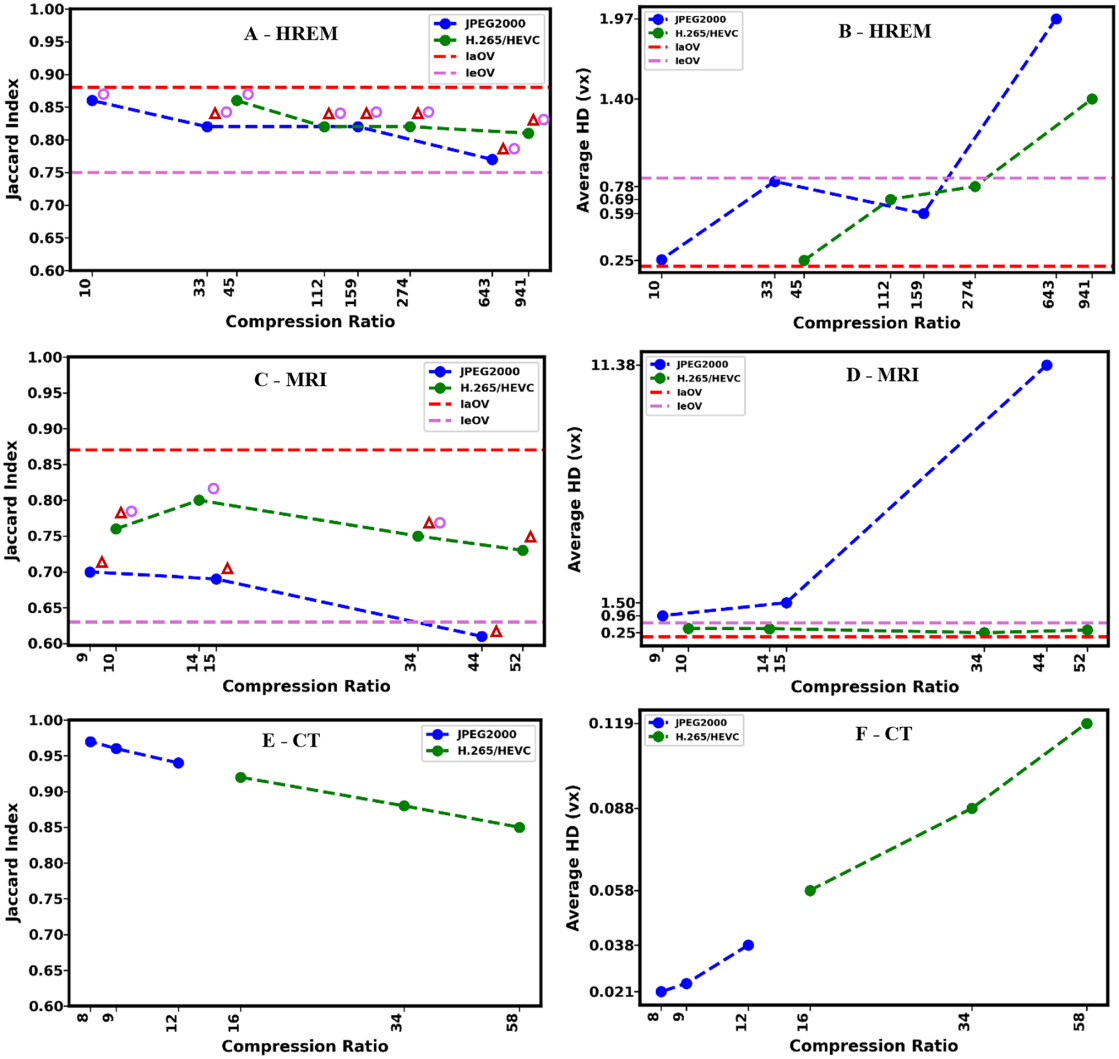
Results of the segmentation performance on lossy compressed images, along with reference intra/inter-observer variabilities, quantified with JI and HD, for each modality. (Top: HREM, middle: MRI, bottom: CT). The red triangle icon indicates that the distribution of Jaccard Indices associated to the presented average value significantly differs (Tukey multiple comparison test) from the intra-observer distribution. The pink circle indicates that the distribution of Jaccard indices associated to the presented average value significantly differs from the inter-observer distribution

Based on the Jaccard indices, we computed intra- and inter-observer reference values for HREM and µMRI for the uncompressed datasets, which are summarized in Table 4 and represented through red and pink dashed lines in Fig. 12. For the intra-observer variability, the same observer segmented the uncompressed datasets twice sequentially after a month, and for the inter-observer variability, two knowledgeable scientists segmented the uncompressed datasets independently. The comparison of these segmentation procedures allowed us to quantify the reproducibility of the segmentation task, and to normalize the results of segmentation after compression with these reference segmentations. In the best case of a single researcher segmenting the data twice, the Jaccard index reaches a maximum value of about 88%, which is even lower if two Segmentors annotate the raw data (maximum JI of only 75 and 63%). We therefore assume that any JI above 75% for HREM and 63% for MRI indicates a clear preservation of the vascular structure, which still allows for the quantitative analysis of the vessel lengths, volumes, and morphologies.

**Table 4:** Reference intra-observer variability (IaOV) and inter-observer variability (IeOV) values, computed as described in the “Intra-observer Variability” and “Inter-observer Variability” sections for each objective score (JI and HD), grouped by image modality (HREM and MRI)

	**HREM**		**MRI**	
**IaOV**	**JI**	**HD**	**JI**	**HD**
	0.880	0.210	0.870	0.080
**IeOV**	**JI**	**HD**	**JI**	**HD**
	0.750	0.840	0.630	0.663

A statistical analysis of the available distributions of Jaccard indices was performed using the Tukey multiple comparison test (confidence level of 95%) to compare all the experimental groups under study. The analysis was executed only for HREM and MRI since those were the modalities involved in the assessment of the manual segmentation task. Due to this, the distributions used to compute the intra-observer variability, and the inter-observer variability were only available for these modalities. In both cases (HREM and MRI), the distributions of Jaccard indices used to compute the intra and inter-observer variabilities showed significant statistical differences between each other, thus clarifying that the dashed lines in Fig. 12, used as delimiting reference levels for our analysis, come from statistically distinct distributions.

As expected, since lossy image compression introduces irreversible changes to the data, most of the evaluated points denoted statistical differences relatively to intra-observer variability (no compression, same observer), except the less compressed HREM files (JPEG2000 10:1, H.265/HEVC 45:1), and MRI H.265/HEVC 14:1. All those points demonstrated the best reported segmentation performance both in terms of Jaccard index and average Hausdorff Distance. In addition, most of the evaluated points also revealed statistical differences relatively to the inter-observer variability (no compression, different observers) except the most compressed H.265/HEVC MRI file, and the JPEG2000 MRI files.

Assuming the inter-observer variability as the minimum threshold for the preservation of vascular morphologies, the segmentation of compressed datasets that leads to statistical differences relatively to the inter-observer distribution, and still presented an average performance (both in terms of JI and HD) greater than the average performance of the inter-observer distribution constituted a valid compression in the context of this study. Whenever this rule does not apply, there is sufficient evidence to advice against the use of such compressions.

Figure 12A, B demonstrate that using lossy compression on the HREM dataset up to 45:1 and 10:1 ratios with H.265/HEVC and JPEG 2000, respectively, resulted in segmentation errors statistically equivalent to the intra-observer variability. For intermediate levels of compression, in which further loss of performance is tolerated, it was verified that H.265/HEVC and JPEG 2000 compressions up to 274:1 and 159:1 induced segmentation errors still below the reference inter-observer variability for both metrics (JI and HD). Compression ratios in the order of 941:1 and 643:1 although close to the inter-observer variability threshold according to the JI, cause significant changes in the segmentations, as shown by the HD curve (Fig. 12B).

A similar analysis considering both the JI and HD results for the MRI dataset indicates that a compression ratio of 52:1 can be achieved without significant impact when comparing to the least compressed H.265/HEVC volume with a compression ratio of 10:1. A compression ratio of 52:1 with H.265/HEVC implies segmentation errors slightly larger than the reference intra-observer variability but inferior to the inter-observer variability score. For this image modality specifically, given its low planar resolution and the very sparse annotations drawn, the JI displayed an overall poor performance in characterizing the impact of compression. This is because the JI constitutes a measure of overlap, with all the wrongly annotated voxels having the same importance. Contrarily, the HD, which considers the magnitude of such errors, displays a different behavior, as depicted in Fig. 12D, which indicates that the least compressed JPEG 2000 volume (compression ratio of 9:1) is already slightly above the reference inter-observer variability. Therefore, was not possible to validate the use of lossy compression of µMRI images for segmentation tasks using JPEG 2000 in this study and this result is coherent with the results of the statistical analysis as all the JPEG2000 compressed MRI images under test did not differ significantly from the inter-observer distribution. This fact demonstrates the robustness and completeness of analyzing the results considering the JI and HD simultaneously.

The results given in Fig. 12 demonstrate the applicability of the Hausdorff Distance in complementing the information provided by the JI. Particularly, while the average JI provides a global overlap measure, the Hausdorff Distance considers the magnitude of the deviations/distances between sets of points or line structures in space. This can be observed in Fig. 12A (HREM), where the average JI between the reference annotation and the H.265/HEVC is the same for the compression ratios of 112:1 and 274:1, but the HD score differs significantly. For the same modality, a decay of the HD scores was verified between JPEG2000 compression ratios of 33:1 and 159:1, while the JI was constant during the same compression ratios. This is due to outlier voxels that were found in a specific portion of the 33:1 compressed dataset, without corresponding annotations in the ground-truth mask at a close spatial location. In this case, given the isotropic voxels, the distance required to traverse along the Z axis (slices) was not particularly penalized when calculating the minimum Euclidean distance between the two points clouds voxel-wise (Eq. 2). As a result, the annotated voxels from the reference mask that were matched to the outliers wrongly annotated in the compressed mask corresponded to points far away along the Z axis but with similar coordinates in the XY plane.

For µMRI, Fig. 12D reveals the presence of errors with significant magnitude in the annotations drawn in JPEG2000 compressed datasets given the sudden jump in the HD curve, as compared to their H.265/HEVC equivalents. In fact, the curve depicted in Fig. 12C provides further evidence about the insufficient use of the JI alone to measure the magnitude of the segmentation errors, since the JI only focuses on the overlap between the annotations without considering the real distance between wrongly annotated points relatively to the points annotated in the reference mask, e.g., in situations of identical segmentation masks up to some offsets.

In the case of the µCT, the results depicted in Fig. 11E, F reveal a consistent decay of the performance of the automated segmentation with the compression ratio for both encoders. Particularly, it was found that for a 12:1 CR, JPEG 2000 was still able to retain 94% of the annotated structures, and more than 85% given a H.265/HEVC 58:1 CR, which translates into an average HD of approximately 0.1 voxel. Overall, we showed that for both HREM and MRI sequences, the variability in the segmentation performance, resulting from the use of lossy compression, was higher than the intra-observer variability but smaller than the inter-observer variability, except for MRI image data encoded with the JPEG 2000 encoder.

**Fig. 13:**
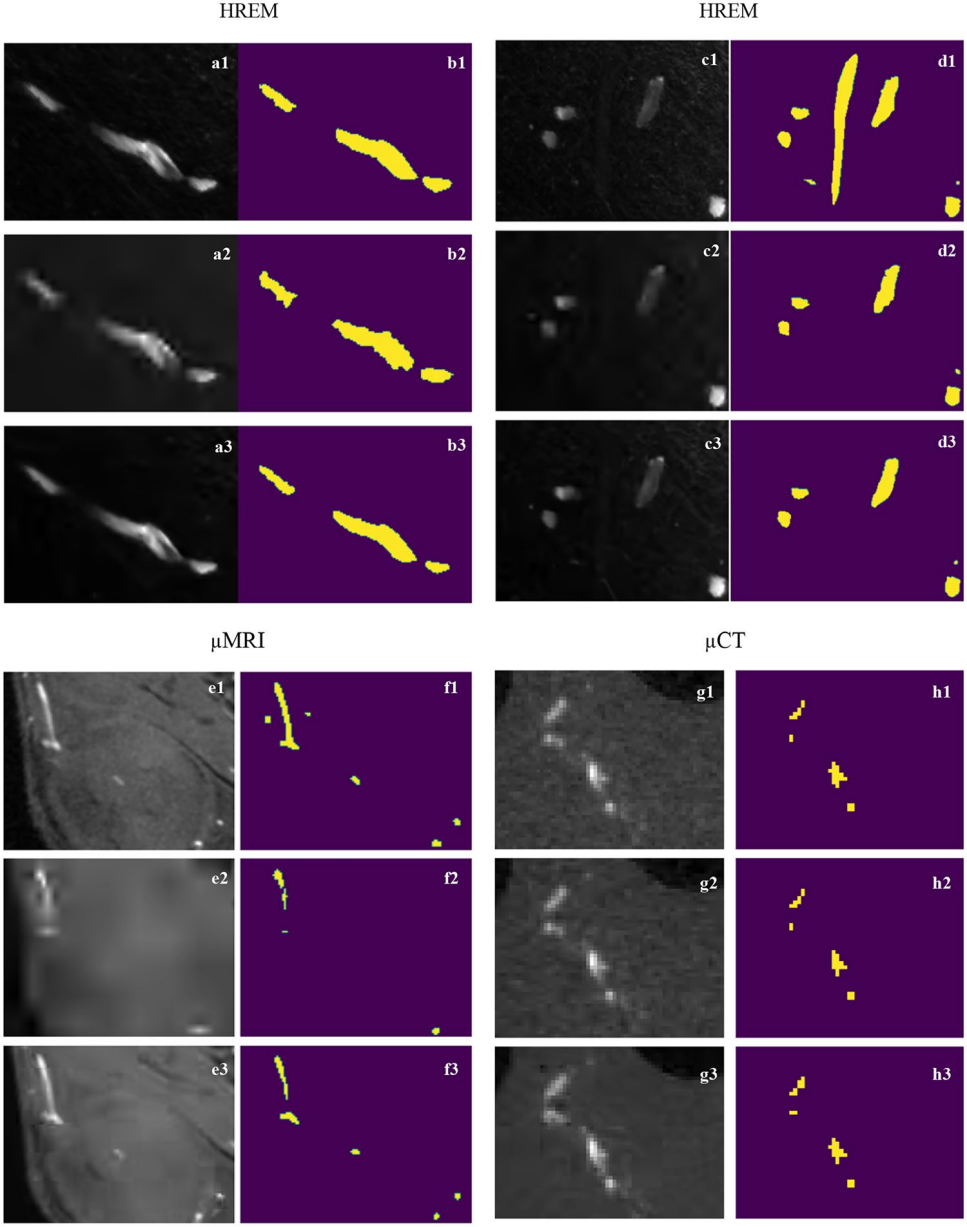
Regions with vessels along with the corresponding annotations extracted from the raw images (sub-index 1), most compressed version with JPEG 2000 (sub-index 2), and most compressed version with H.265/HEVC (sub-index 3)

Figure 13 showcases examples of the use of the highest compression ratios under test for both encoders for each image modality. Particularly, the region depicted in Fig. 13a2, a3 demonstrates the robustness of both encoders in preserving the HREM texture information in the case of a prominent cross section of a vessel appearing in a region with a flat homogeneous background. The high planar (xy) resolution of HREM images ensures that the size of the vessels sections is typically larger than the coding block sizes used by H.265/HEVC (from 4 × 4 to 64 × 64 pixels); this can also explain why artefacts are not observed due to blocking effect in the H.265/HEVC compressed images. However, some ringing artefacts on the JPEG 2000 compressed version can be seen at the regions of high-frequency content, corresponding to the background/foreground interface (Fig. 13a2). A different scenario is presented in the second group (Fig. 12c1, c2, c3) consisting of a vascular cross section with poor contrast relative to the tumor tissue. Since no steep edges separate the foreground from the background, both encoders smooth out the local texture in a process similar to a low-pass filter that makes the segmentation of the objects even more difficult after compression.

Figure 13e, g demonstrate that the lower spatial resolution of µCT and µMRI sequences that, combined with challenging image statistics, make the images more prone to compression artefacts, as depicted by the block effects evidenced after H.265/HEVC compression (Fig. 13e3, g3). For direct visual comparison between the most compressed µCT images with JPEG2000 (Fig. 13g2) and H.265/HEVC (Fig. 13g3), it should be noted that they are encoded with a significantly different number of bits (Fig. 9).

**Fig. 14:**
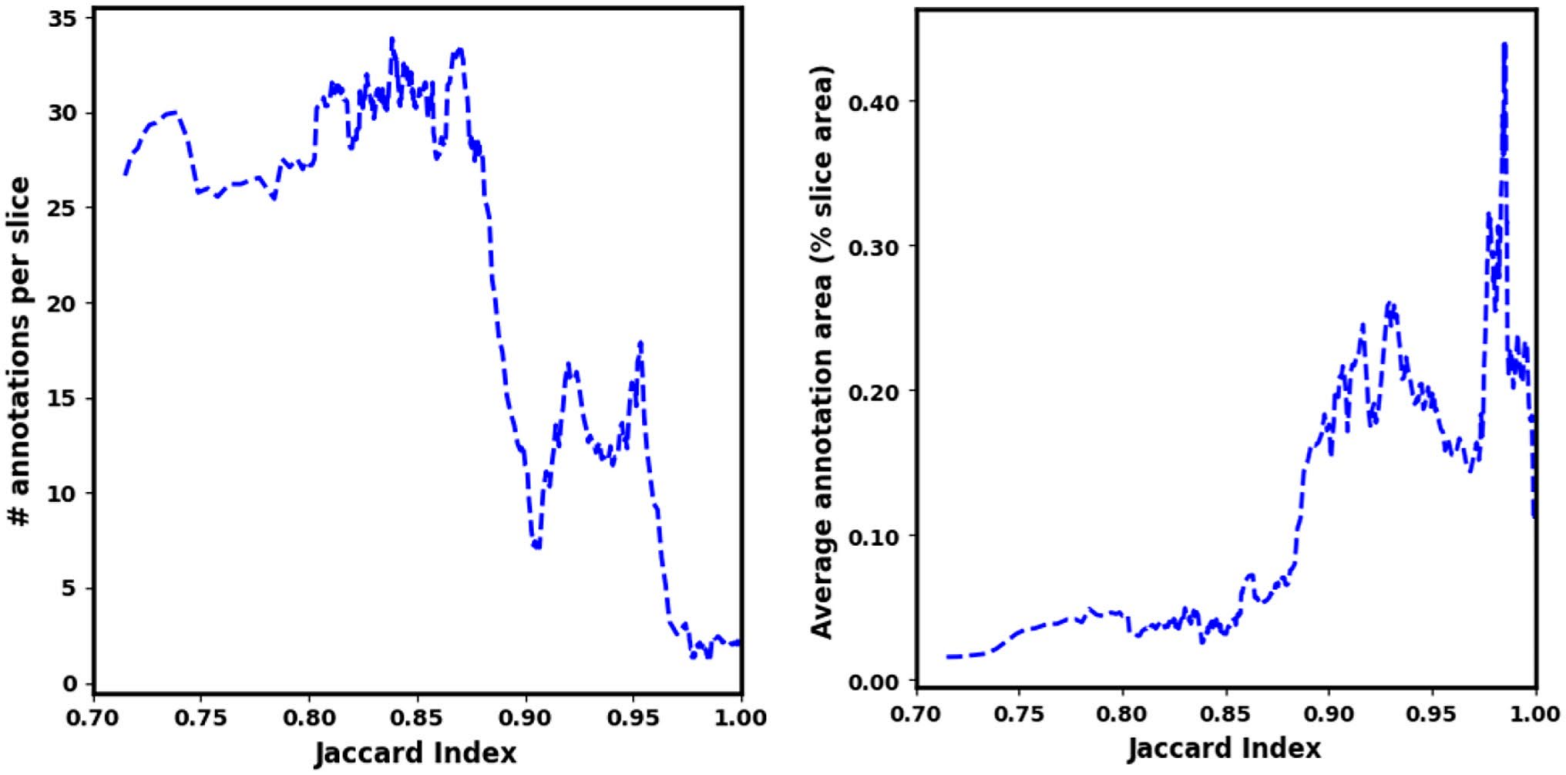
Performance assessment of the HREM segmentation between time-points t_0_ and t_1_ on the raw dataset, as a function of the number of annotations per analyzed slice (left) and the average annotation area per analyzed slice (right)

Although the slices of both modalities evidence sparse distribution of vessel clusters, most of the vessels’ cross-sections imaged by MRI appear as single-dots, which are particularly challenging to be segmented, even in a compression-free scenario. In contrast, the HREM slices often included larger continuous vascular regions that facilitates the segmentation task and make it more robust to errors related to fatigue or any other human-related condition. Following this idea, we showed that the size of the annotated regions presented a predictor of the complexity of the segmentation task, since larger continuous structures are typically easier to identify and track slice-wise. Figure 14 shows the JI between the first and second HREM segmentations of the raw data (i.e., compression free) against the number of annotated vessels and the average annotation area normalized by the slice area. The behaviors of these curves demonstrate that the segmentation task is simplified by the presence of fewer but larger structures.

## Discussion

A first comparison of the performance of the two encoders showed that H.265/HEVC delivered reconstructions of higher quality for a given compression ratio, which highlights its ability to represent the image information in a more compact way than JPEG 2000 (Fig. 9). This can be explained with the fact that H.265/HEVC is able to exploit the volumetric redundancy through motion estimation, thus leading to more compact bitstreams than JPEG2000, as consistently verified in this study. However, by operating slice-wise without any inter-slice coding dependency, JPEG 2000 can be used to encode dense volumetric sequences made of thousands of slices in parallel, with potential significant savings in the total encoding time. JPEG 2000 offers an additional advantage over H.265/HEVC, which is related to its fine rate-control mechanism. The rate-distortion curves confirm that the rate-control implemented by the JPEG 2000 encoder is more accurate given the low magnitude of the corresponding error bars. This aspect can be particularly relevant in the case of volumetric biomedical images as the coding mechanism allows quality-control on a slice-basis, which can be used according to the importance of the data at specific slice indices.

The evaluation of the compressed images using a texture-based descriptor confirmed the reduction of the details, and therefore of the image complexity, with the increase of the compression ratio (Table 2). Particularly, for the maximum compressed versions of each modality (34:1 for CT, 52:1 for MRI, and 274:1 for HREM) the reduction of the texture descriptor value relatively to the corresponding uncompressed images was of 1.9%, 10%, and 6.3% respectively. Since the computation of the descriptor was exclusively performed around annotated regions, these values demonstrate the robustness of the encoders in preserving the relevant morphological features even considering the significant compression ratios.

The definition of a minimum overlap value $t$ used to identify the valid slices $V$ where the intra-observer agreement was above 70% in a compression-free scenario, revealed that the JI is a poor description of the segmentation performance. In fact, it was shown that a JI value of 0.7 correlates with the preservation of most structural details, particularly in cases where the foreground objects (vessels) are extremely thin. For example, in cases of vessels of a width of 2 pixels, missing only 1 pixel while annotating the structures will result in a decay of the JI to 0.5. In such cases, a pre-processing step that skeletonizes the vessel structure would help to normalize the annotations, prior to the computation of the JI.

Assuming the JI of the inter-observer variability is the minimum threshold for the preservation of vascular morphologies and volume, it was concluded that lossy compression schemes were applicable to (i) the HREM dataset with compression ratios up to 274 for H.265/HEVC and up to about 159 for JPEG2000, (ii) and to the MRI dataset with compression ratios up to 52 for H.265/HEVC. The µCT dataset was found to be compressible up to 34 times with H.265/HEVC and slightly more than 12 times with JPEG 2000, while maintaining the JI approximately at 0.90 or more. In metric terms, these compression setups originated average segmentation errors not greater than 0.1 voxel. Based on the previously published CMI feasibility study [11] where the morphological changes of the murine vasculature was assessed upon acquired resistance of in total 10 tumors, it was concluded that the data volume for HREM can be reduced from 510 GB to 1.9 GB (10 datasets × 51 GB divided by 274), the data volume for µMRI can be reduced from 360 MB to about 7 MB (10 datasets × 36 MB divided by 52), and the data volume for µCT can be reduced from 3 GB to 88 MB (10 datasets × 300 MB divided by 34) to still preserve the biomedically relevant vascular morphology and allow the same conclusions. *In total, considering the test volumes used in this study, there was a reduction of the data load from 513 GB to approximately 2 GB — a 256-fold reduction, while still allowing the quantitative assessment of the tumor vasculature across all modalities.*

All the above studies are supported by objective metrics that can be adopted by any bioimaging team interested in quantitatively analyzing the impact of lossy compression on a specific bioimage processing pipeline. The proposed framework can also be used to evaluate the performance of one-to-multiple experts analysts for a given task, independent of lossy compression.

We have shown with our method that the JI alone is not sufficient to quantify segmentation performance. Applying the Hausdorff distance together with the JI allowed us to overcome these shortcomings, which is supported by the multiple comparisons statistical analysis of the Jaccard index distributions. Thus supported the conclusions drawn only after considering the HD results too, as particularly well depicted in Fig. 12C/D. Due to the discrepancy of data volume among the three modalities, the significance of the main findings should be put into perspective since most of the data volume is contributed in this study by HREM. In future, the authors will focus on extending the proposed framework to other experiments, involving different modalities but, preferably, with a more balanced multi-modal dataset. Another limitation of the present study corresponds to the fact that the inter-observer variability was exclusively based on uncompressed data; ideally, the inter-observer variability would be quantified when moving from the original to the compressed domain. However, this was not feasible since manual segmentations were very time consuming. Given these conditions, we decided to define the inter-observer variability using the best available data (uncompressed), to isolate the potential impact of compression distortions.

To the best of our knowledge, this is the first attempt to perform a thorough assessment on the impact of lossy compression using standard compression technology in a segmentation problem that involves multimodal imaging across scales in a pre-clinical context.

It is important to note that the achieved 256-fold reduction in data volume does not only tremendously alleviate the data storage and data transmission challenges, but can also facilitate data visualization: specifically, for the HREM datasets, manual segmentation was not trivial to handle in terms of computing power. This holds true for research laboratories in general, and specifically for imaging facilities that produce GBs of data daily, which need to be stored and shared. Based on a discussion with Euro-BioImaging, a European research infrastructure consortium, we believe that in Europe alone, there are about 1200 bioimaging facilities that we estimate to produce PBs of data per year [41]. There are currently no standards and recommendations on how preclinical and biological imaging data across modalities should be archived and stored in terms of compression. Specifically, for imaging research infrastructures and facilities, well-defined data compression schemes will help to facilitate data storage and sharing, and to efficiently manage the big-data regime in biomedical imaging.

## Conclusions

In this work, we (i) compared achievable compression rates and data storage reductions for HREM, µCT, and µMRI using two standardized lossy encoding schemes (JPEG2000 and H.265/HEVC), and (ii) assessed a maximal compression rate that ensures preservation of relevant image content (in this case, tumor vasculature) comparatively to the uncompressed raw data, for each encoder. Compression rates and volume reductions of up to 256 were achieved for the HREM datasets for the H.265/HEVC compression that, in general, reached higher compression ratios when compared to JPEG2000. The identification of compression ratios that still preserve the structural integrity of the vasculature and allow for quantitative analyses was based on several qualitative and quantitative assessments. After visual comparison of all segmented vessels for each imaging modality, the Jaccard index complemented by the Hausdorff Distance served as a measurement for the data similarity between decompressed and uncompressed datasets. Importantly, we observed very large variability even for the reference data that were produced by manual segmentation. Comparing the Jaccard index values computed on segmentations of compressed and uncompressed datasets allowed us to define a minimum acceptable Jaccard index. This analysis was achieved by comparing manual segmentations of the same uncompressed dataset from two independent Segmentors (inter-observer variability) and from a single Segmentor at different time points (intra-observer variability). This procedure allowed to normalize the segmentation results after compression relatively to such baselines, and to set a Jaccard index of about 0.7 as a measurement of good structural preservation in a compression-free scenario.

We believe the results of this work and the recommendations about which compression algorithms (and at which compression level) can be used safely can be useful to the biomedical imaging community and bioimaging facilities and represents a first step towards highlighting and defining compression schemes for improved storage, sharing and analysis of (compressed) big imaging data, both for microscopy and preclinical modalities.

## Data Availability

The data that support the findings of this study are available upon reasonable request.
