# Correction to: Lossy Image Compression in a Preclinical Multimodal Imaging Study

**DOI:** 10.1007/s10278-023-00879-w

**Published:** 2023-07-19

**Authors:** Francisco F. Cunha, Valentin Blüml, Lydia M. Zopf, Andreas Walter, Michael Wagner, Wolfgang J. Weninger, Lucas A. Thomaz, Luís M. N. Tavora, Luis A. da Silva Cruz, Sergio M. M. Faria

**Affiliations:** 1 Instituto de Telecomunicações, Morro do Lena—Alto do Vieiro, Leiria, Portugal; 2 University of Coimbra, Coimbra, Portugal; 3 Vienna BioCenter Core Facilities GmbH, 1030 Vienna, Austria; 4 Ludwig Boltzmann Institute for Experimental and Clinical Traumatology Vienna, Vienna, Austria; 5 Centre of Optical Technologies, Aalen University, Aalen, Germany; 6 Institute of Applied Research, Aalen University, Aalen, Germany; 7 Division of Anatomy, Center for Anatomy & Cell Biology, Medical University of Vienna, Vienna, Austria; 8 School of Technology and Management, Polytechnic of Leiria, Morro do Lena—Alto do Vieiro, Leiria, Portugal; 9 Department of Electrical and Computer Engineering, University of Coimbra, Coimbra, Portugal; 10 Instituto de Telecomunicações, University of Coimbra, Coimbra, Portugal

**Correction to: Lossy Image Compression in a Preclinical Multimodal Imaging Study** **https://doi.org/10.1007/s10278-023-00800-5**

The corresponding author for this paper should be Andreas Walter; details are below.

Andreas Walter

Centre of Optical Technologies, Aalen University, Aalen, Germany

andreas.walter@hs-aalen.de

The original article has been corrected.

